# Structural and functional characterization of the endogenous agonist for orphan receptor GPR3

**DOI:** 10.1038/s41422-023-00919-8

**Published:** 2024-01-30

**Authors:** Geng Chen, Nico Staffen, Zhangsong Wu, Xinyu Xu, Jinheng Pan, Asuka Inoue, Tingyi Shi, Peter Gmeiner, Yang Du, Jun Xu

**Affiliations:** 1 Kobilka Institute of Innovative Drug Discovery, Shenzhen Key Laboratory of Steroid Drug Discovery and Development, School of Medicine, Chinese University of Hong Kong, Shenzhen, Guangdong China; 2 Department of Chemistry and Pharmacy, Medicinal Chemistry, Friedrich-Alexander University Erlangen-Nürnberg, Nikolaus-Fiebiger-Straße 10, Erlangen, Germany; 3 Beijing Advanced Innovation Center for Structural Biology, School of Pharmaceutical Sciences, Tsinghua University, Beijing, China; 4 Mass Spectrometry & Metabolomics Core Facility, Biomedical Research Core Facilities, Westlake University, Hangzhou, Zhejiang China; 5 Graduate School of Pharmaceutical Sciences, Tohoku University, Sendai, Japan; 6 Department of Molecular and Cellular Physiology, Stanford University School of Medicine, Stanford, CA USA

**Keywords:** Cryoelectron microscopy, Molecular modelling

Dear Editor,

GPR3 is a class A orphan G protein-coupled receptor (GPCR) exhibiting broad expression across various brain regions including the hypothalamus, hippocampus, and cortex, as well as in peripheral tissues such as liver and ovary.^1,2^ Previous studies have highlighted the critical roles of GPR3 in regulating a diversity of physiological functions, including neurite outgrowth/neuronal survival, neuropathic pain, and oocyte maturation.^2^ Intriguingly, in vivo studies using Alzheimer’s disease (AD) mouse models provide evidence showing that GPR3 can regulate the activity of γ-secretase and mediate the amyloidogenic proteolysis of the amyloid precursor protein (APP).^3^ This finding positions GPR3 as a potential therapeutic target for the treatment of AD.

GPR3 has been characterized as a constitutive activator of adenylate cyclase through coupling to the heterotrimeric Gs protein.^4^ The constitutive activity is notably similar to that of the self-activated orphan receptor GPR52,^5^ surpassing other Gs-coupled receptors like β_2_AR (Supplementary information, Fig. S1a, b). Recently, it has been discovered that the high intrinsic activity of GPR3 plays a crucial role in regulating adipose thermogenesis in response to cold-induced lipolysis.^6^ Two potential scenarios may explain the high constitutive activity of GPR3^4^: basal coupling with Gs in the absence of a ligand, or stimulation by a ubiquitous ligand (Fig. 1a). Given the GPR3 sequence similarity to cannabinoid, free fatty acid, sphingosine-1-phosphate (S1P) and lysophosphatidic acid (LPA) receptors, and considering the properties of endogenous ligands of these receptors (Fig. 1b, c), it has been postulated that the ligand of GPR3 might be a membrane-bound or membrane-derived lipid.^4^ Although some studies have suggested certain lysophospholipids (such as S1P) as potential agonists of GPR3, the conclusion remains controversial, as the activation effect of these lipids were not reproduced in other studies.^7^

**Fig. 1:**
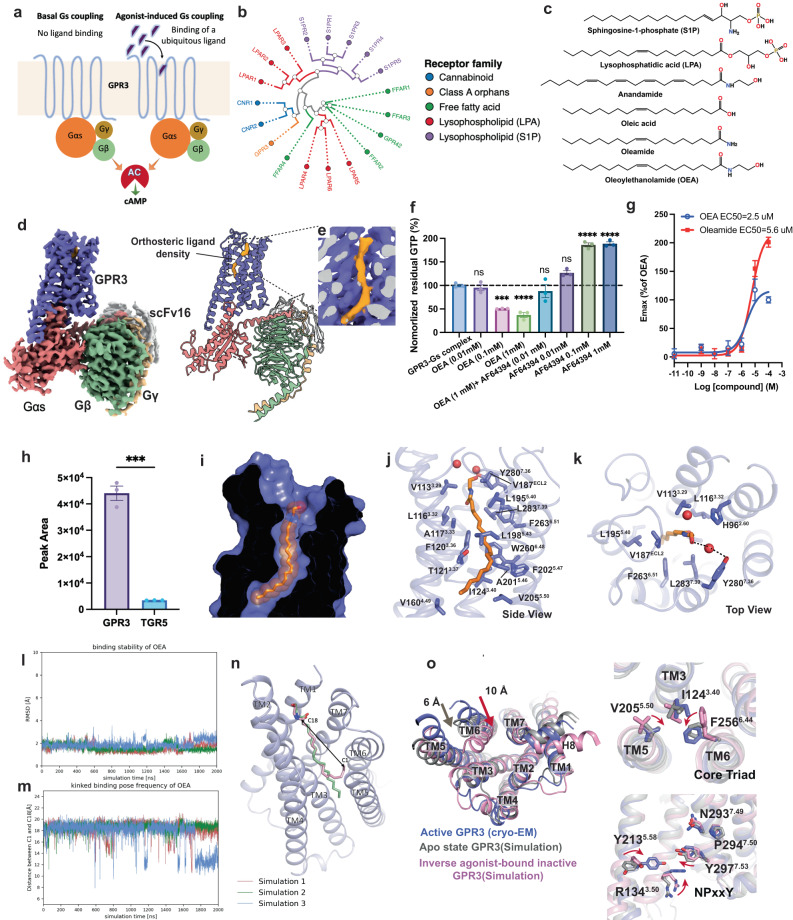
Structural and functional characterization of the endogenous agonist for GPR3. **a** A schematic illustration of two proposed mechanisms for the high constitutive activity of GPR3. **b** Phylogenetic tree of the orphan GPR3, cannabinoid receptors, free fatty acid receptors, LPA receptors and S1P receptors. **c** Chemical structures of different lipid molecules or lipid metabolites. **d** Cryo-EM map and structural model of GPR3–Gs signaling complex. **e** Enlarged density map of the lipid-like molecule located in the extracellular orthosteric pocket of GPR3. **f** Activity of OEA and inverse agonist AF64394 on GPR3 measured by in vitro GTP turnover assay using purified native GPR3–Gs complex in detergent micelles. Error bars denote means ± SEM. Statistical analyses were performed using the ordinary one-way ANOVA. ***P  <  0.001, ****P  <  0.0001, ns (not significant). **g** Concentration response curves of OEA and oleamide measured by cell-based cAMP-Glo Sensor assay. Error bars denote means ± SEM of three independent experiments. **h** MS of OEA in the GPR3–Gs and TGR5–Gs complexes. Data are shown as means ± SEM from three independent experiments. Statistical analyses were performed using the ordinary one-way ANOVA. ***P   <  0.001. **i** Cross-section of GPR3 is shown, with the interior in black. OEA, binding to the tunnel-like orthosteric pocket, is shown in orange sphere. **j**, **k** Detailed interactions between OEA and the orthosteric pocket from side (**j**) and top (**k**) views. **l**–**n** MD simulations of OEA binding to GPR3. RMSD for OEA in comparison to its starting conformation (**l**). The distance of C1 and C18 of the alkyl chain as a measurement for the frequency of a kinking motion (**m**). Two representative MD snapshots of GPR3 binding OEA in either a straight or a kinked binding pose (**n**). **o** Structural comparison of overall structures of GPR3 in the inactive inverse agonist-bound state (pink), apo state (gray) and active state (slate). Key conformational changes including the TM6 movement, rearrangement of the core triad and NPxxY motif are highlighted with red arrows.

To elucidate the molecular mechanism underlying the high constitutive activation of GPR3, here, we report a cryo-EM structure of the GPR3–Gs signaling complex at a global nominal resolution of 3.03 Å (Fig. 1d; Supplementary information, Figs. S1, S2, Table S1). We used both scFv16^8^ and nanobody Nb35^9^ to stabilize the complex for cryo-EM analysis. However, most particles lacked density of Nb35. Consistent with this, the final sample contained a weak band for Nb35 as indicated by the SDS-PAGE analysis (Supplementary information, Fig. S1c). Because the map without Nb35 exhibited better local resolution in the receptor region (Supplementary information, Fig. S2a, b), we used this map for model building and subsequent analysis.

The 3.03 Å map allowed us to unambiguously build the molecular structure of most regions within the signaling complex (Fig. 1d). Interestingly, we observed a lipid-like molecule bound to the orthosteric pocket of GPR3, which was co-purified with the receptor from insect cell membranes (Fig. 1e). Based on the density shape and previous reports, we initially suspected that the ligand might be a lysophospholipid (Fig. 1c). However, in vitro GTP turnover assay using purified native GPR3–Gs complex (Supplementary information, Fig. S3a) did not show an agonist effect on GPR3 for several lysophospholipids including S1P, LPA and lysophosphatidylserine (LPS) (Supplementary information, Fig. S3b). Similar results were observed from cell-based signaling assay (Supplementary information, Fig. S3c). Notably, high concentrations of LPA showed strong inhibitory effects on receptor activity in GTP turnover assay (Supplementary information, Fig. S3b), possibily due to disruption of detergent micelles and protein denaturation. In line with the functional data, structural comparison with S1P receptor 1 (S1PR1) and LPA receptor 1 (LPAR1) revealed that there is a lack of polar or charged residues in the extracellular surface of GPR3 that may interact with the large phosphate moiety of lysophospholipids (Supplementary information, Fig. S3d). Recent functional studies suggested that the N-terminus of GPR3 is essential for the constitutive activity.^6^ However, we did not observe clear electron density for the N-terminus in our cryo-EM map, suggesting that, unlike the helix conformation observed in S1PR1 and LPAR1, this region in GPR3 is highly flexible (Supplementary information, Fig. S3d).

Subsequently, we explored the possibility that the ligand could be certain lipid metabolites with smaller polar heads, such as free fatty acids or related bioactive amides. Manual testing of a number of bioactive fatty acids or amides led us to identify oleic acid and its derivatives, oleamide and oleoylethanolamide (OEA), as potential candidates that could be well-modeled into the density (Fig. 1c; Supplementary information, Fig. S3e). Among them, OEA exhibited the best fit into the density map (Supplementary information, Fig. S3e). Importantly, we observed a significant stimulating effect of OEA at concentrations of 0.1 mM and 1 mM in the GTP turnover assay (Fig. 1f). Additionally, OEA showed no effect on Gs, indicating its selective activity on the receptor (Supplementary information, Fig. S4a). Moreover, the activity of OEA was antagonized by the previously reported GPR3 inverse agonist AF64394 (Fig. 1f).^10^ In contrast to OEA, we did not observe significant activity for oleamide and oleic acid, as well as a series of free fatty acids having similar structure to oleic acid including linoleic acid, stearic acid, palmitic acid, arachidonic acid, 9-HSA and EPA (Supplementary information, Fig. S4b, c). Similar to LPA, most of these free fatty acids showed inhibitory effects on the basal activity of GPR3 when applied at high concentrations (> 0.1 mM) (Supplementary information, Fig. S4c).

To confirm the agonist effect of OEA, we also expressed GPR3 in the presence of AF64394 and obtained the monodisperse GPR3 in apo form by gradually removing AF64394 during purification process (Supplementary information, Fig. S5a). Notably, significant GTP turnover was detected using the purified apo-form GPR3, indicating basal coupling between GPR3 and Gs (Supplementary information, Fig. S5b). Consistent with the results obtained using GPR3–Gs compelx, the addition of OEA futher increased the GTP turnover efficacy, thereby demonstrating its agonist effect. No stimulation effect was observerd for other lipid molecules (Supplementary information, Fig. S5b). Additionally, we detected direct interaction between OEA and GPR3 using surface plasmon resonance (SPR) (Supplementary information, Fig. S5c).

We further performed cell-based signaling assay to validate the agonist effect of OEA on GPR3. In accordance with the GTP turnover assay, cAMP Glo-Sensor assay also showed activation of GPR3 by OEA with an EC_50_ value of 2.5 μM (Fig. 1g). Unexpectedly, we found that oleamide can also activate GPR3 in the cell-based assay with higher efficacy but lower potency (EC_50_ of 5.6 μM) than OEA (Fig. 1g). This is probably because oleamide binding requires the native environment. Consitent with GTP turnover assay, we did not observe significant agonist effect for most of the free fatty acids in the cell-based assay except for the weak activity of palmitic acid (Supplementary information, Fig. S4d).

To further validate OEA as the observed ligand in our cryo-EM map, we performed a mass spectrometry (MS)-based analysis of the detergent-solubilized protein sample used for structural studies. As expected, we observed significant enrichment of OEA in the GPR3 sample compared to the control receptor TGR5, which was purified in the same way from Sf9 cells while showing much lower OEA enrichment (Fig. 1h; Supplementary information, Fig. S6). Taken together, these results suggest that the observed density in the orthosteric pocket most likely represents OEA, which serves as a candidate of GPR3 endogenous agonist and may contribute to the high constitutive activity of GPR3 (Supplementary information, Fig. S1a, b). Notably, OEA is structurally an endocannabinoid-like metabolite (Fig. 1c), consistent with the fact that GPR3 is evolutionarily most close to the cannabinoid receptors (Fig. 1b). Moreover, recent studies have shown that cold-induced lipolysis can trigger GPR3 transcription to drive adipose thermogenesis, and that dietary fat can potentiate GPR3-depedent thermogenesis.^6^ It is plausible that these processes contribute to the production of OEA and other bioactive lipid amides to enhance GPR3 functionality.^11^

Further structural analysis revealed that the OEA binding pocket is composed of TM3, 5, 6 and 7, which creates a highly hydrophobic tunnel by a series of non-polar residues (Fig. 1i–k), allowing the insertion of the long alkyl chain of OEA. Such a tunnel pocket has also been observed for S1P receptors and an orphan receptor GPR88.^12,13^ Mutagenesis studies demonstrated the importance of these tunnel residues for receptor function; for example, alanine replacement of large aromatic residues (i.e., H96^2.60^A, F263^6.51^A, W260^6.48^A and F202^5.47^A) or mutation of small hydrophobic residues to large aromatic residues (i.e., L283^7.39^F, L116^3.32^F, T121^3.37^F and I124^3.40^F) significantly reduced the basal activity of GPR3 or OEA-induced receptor activation (Supplementary information, Fig. S7a–c). Noteworthily, these mutations had little effect on receptor expression (Supplementary information, Fig. S7a). The lack of direct polar interaction between the OEA head group and GPR3 was compensated by the presence of two potential water molecules on top of OEA, which creates a relatively hydrophilic environment (Fig. 1k; Supplementary information, Fig. S7d). One of the water molecules mediates the hydrogen-bonding interactions between Y280^7.36^ and the oxygen of the OEA hydroxyethyl group (Fig. 1k). In accordance with the structural model, Y280^7.36^A mutant showed reduced receptor activity (Supplementary information, Fig. S7b, c). We also observed that deletion of the N-terminus (N36) almost abolished receptor activity, suggesting its important role in GPR3 signaling (Supplementary information, Fig. S7b, c). This result is similar to a recent study;^6^ however, the molecular mechanism remains unclear due to the lack of density for the N-terminus.

To further validate the binding mode of OEA, we performed three independent 2-μs runs of molecular dynamics (MD) simulations. The results showed that OEA maintains a relatively stable conformation through the simulation time with a root-mean-square-deviation (RMSD) of 1.7 Å (Fig. 1l). Some of the outliers are caused by occasional kinking motions from the tail of the alkyl chain (Fig. 1m, n). These observations align well with the relatively weak interactions between the tail and GPR3 (Fig. 1j). Notably, the MD simulations on oleamide revealed higher frequency of RMSD outliers and the kinking motion of the tail than OEA (Supplementary information, Fig. S8a–c), further supporting the notion that OEA is the most likely candidate for the endogenous ligand observed in the cryo-EM map.

To gain insights into the conformational transitions of GPR3 upon agonist binding and G protein coupling, we conducted MD simulations based on our cryo-EM structure. Taking advantage of metadynamics, the calculations converged to a stable conformation after removal of ligand and G protein (Supplementary information, Fig. S9). Interestingly, this apo model shares both similarities with the active state and a typical inactive state of class A GPCRs. Using the identical metadynamics protocol, we have shown that the apo state is very similar to an inverse agonist-bound inactive state for other GPCRs (β_2_AR, MOR, M2R, GPR88).^13^ As shown in Fig. 1o, almost no conformational changes in the conserved V^5.50^I^3.40^F^6.44^ and N^7.49^P^7.50^xxY^7.53^ motifs occurred compared to the active state cryo-EM structure, while an inward shift of TM6 by 6 Å occurred along with breaking of the Y^5.58^Y^7.53^ motif, both hallmarks of receptor inactivation. In addition, a downward rotation of the D^3.49^R^3.50^Y^3.51^ motif can be observed, which is typical for class A GPCRs in an inactive state. The model indicates that GPR3 in its apo state resides in a conformation between active and inactive states, which may be favorable for the basal coupling with Gs and contribute to the observed basal activity of GPR3 (Supplementary information, Figs. S1a, S5b). A previously published model of GPR3 bound to the inverse agonist AF64394 obtained by MD simulations^14^ shows that GPR3 may undergo conformational changes typical for other class A GPCRs including a 10 Å movement of TM6 and rearrangements of the conserved micro-switches (Fig. 1o). However, this inactive state requires stabilization by an inverse agonist and appears to be different from the apo state.

In the G protein-coupling interface, the C-terminal α5 helix of Gαs inserts into the receptor core, forming extensive hydrophilic and hydrophobic contacts with residues in TM3, TM5, TM6, TM7 and ICL2 (Supplementary information, Fig. S10). In general, the GPR3–Gs complex displays high similarity in the receptor–G protein interactions compared with other reported structures of class A GPCR–Gs complexes, such as V2R and β_2_AR,^9,15^ suggesting a common mechanism for G protein coupling.

In summary, we determined the cryo-EM structure of the GPR3–Gs signaling complex, which revealed an unknown endogenous ligand density within the orthosteric pocket of GPR3. Through a comprehensive approach involving molecular modeling, functional assays, MS and MD simulations, we identified the lipid derivative OEA as a promising candidate for the endogenous agonist of GPR3. Additionally, our analysis of the active state structure in comparison to the simulated apo state and an inverse agonist-bound inactive state models provided valuable insights into the activation mechanism of GPR3. These findings collectively support the notion that the high constitutive activity of GPR3 arises from the combined effects of stimulation by an ubiquitous ligand and its basal coupling with Gs (Fig. 1a). Altogether, our study establishes a crucial structural basis for unraveling the signaling mechanism of GPR3 and offers a valuable template for future endeavors in structure-based discovery of small-molecule drugs targeting GPR3.

### Supplementary information

Supplementary information

## Data Availability

The 3D cryo-EM density map of the OEA-bound GPR3–Gs–scFV16 complex has been deposited in the Electron Microscopy Data Bank under accession code EMD-38015. The atomic coordinates for the atomic model of the OEA-bound GPR3–Gs–scFV16 complex generated in this study have been deposited in the Protein Data Bank under accession code 8X2K.

## References

[1] Iismaa TP (1994). Genomics.

[2] Morales P, Isawi I, Reggio PH (2018). Drug Metab. Rev.

[3] Huang Y (2015). Sci. Transl. Med..

[4] Eggerickx D (1995). Biochem. J..

[5] Lin X (2020). Nature.

[6] Sveidahl Johansen O (2021). Cell.

[7] Yin H (2009). J. Biol. Chem..

[8] Maeda S (2018). Nat. Commun..

[9] Rasmussen SG (2011). Nature.

[10] Jensen T (2014). Bioorg. Med. Chem. Lett..

[11] Schwartz GJ (2008). Cell Metab..

[12] Liu S (2022). Nat. Commun..

[13] Chen G (2022). Nat. Commun..

[14] Bharathi, Roy KK (2022). J. Biomol. Struct. Dyn..

[15] Wang L (2021). Cell Res..

