# Supplementary material for: Structural and functional characterization of the endogenous agonist for orphan receptor GPR3
Source: Cell Res. 2024 Jan 30;34(3):262–5. doi: 10.1038/s41422-023-00919-8 (PMC10907609; doi:10.1038/s41422-023-00919-8)
Supplement: Supplementary file 1 — Supplementary information [file 41422_2023_919_MOESM1_ESM.pdf]

Supplementary information

Structural and functional characterization of the endogenous agonist for orphan receptor GPR3

Geng Chen[1,#], Nico Staffen[2,#], Zhangsong Wu[1,#], Xinyu Xu[3], Jinheng Pan[4], Asuka Inoue[5], Tingyi Shi[1] , Peter Gmeiner[2,*], Yang Du[1,*], Jun Xu[1,6,*]

[1]Kobilka Institute of Innovative Drug Discovery, Shenzhen Key Laboratory of Steroid Drug Discovery and Development, School of Medicine, Chinese University of Hong Kong, Shenzhen, Guangdong, China.

[2]Department of Chemistry and Pharmacy, Medicinal Chemistry, Friedrich-Alexander University Erlangen-Nürnberg, Nikolaus-Fiebiger-Straße 10, Erlangen, Germany.

[3]Beijing Advanced Innovation Center for Structural Biology, School of Pharmaceutical Sciences, Tsinghua University, Beijing, China.

[4]Mass Spectrometry & Metabolomics Core Facility, Biomedical Research Core Facilities, Westlake University, Hangzhou, China.

[5]Graduate School of Pharmaceutical Sciences, Tohoku University, Sendai, Japan

[6]Department of Molecular and Cellular Physiology, Stanford University School of Medicine, Stanford, CA, USA.

# These authors contributed equally: Geng Chen, Nico Staffen, Zhangsong Wu.

*Corresponding Authors: Peter Gmeiner peter.gmeiner@fau.de; Yang Du yangdu@cuhk.edu.cn ; Jun Xu junxu@stanford.edu

Materials and Methods

Expression and purification of GPR3

GPR3 was expressed in insect cells using the Bac-to-Bac baculovirus expression system. Briefly, WT Homo sapiens full length GPR3(NM_005281.3) was cloned into pFastbac1 vector. To increase protein expression and stability, an N-terminal HA signal peptide, followed with the Flag epitope, a 3C protease site, and a BRIL fusion protein were inserted before the GPR3 sequence. The construct was transformed to DH10bac cell and the recombinant bacmid was extracted to produce baculovirus for GPR3 expression in Sf9 (Spodoptera frugiperda) cells. Sf9 cells were infected with the baculovirus at a density of 4×10^6 cells per ml and were collected after 48 hours.

For expression and purification of monodisperse GPR3, 1 µM inverse agonist AF64394 was added during expression. Cells were collected after 48 hours and then lysed in the lysis buffer 10 mM HEPES, pH 7.5, 10 µM AF64394, 1 mM EDTA, 4 mg/ml iodoacetamide, 2.5 µg/ml leupeptin, 0.16 mg/ml Benzamidine for 1 hour at room temperature. Then the cells were centrifuged at 12,000g for 1 hour to collect the membrane and the membrane was solubilized in a buffer 20 mM HEPES, pH 7.5, 100 mM NaCl, 10 µM AF64394, 1% LMNG (NG310 Anatrace), 0.1% CHS (CH210, Anatrace), 4 mg/ml iodoacetamide, 2.5 µg/ml leupeptin, 0.16 mg/ml Benzamidine for 2 hours. Extracted GPR3 protein was further purified by M1 flag resin affinity chromatography and finally eluted by elution buffer 20 mM HEPES, pH 7.5, 100 mM NaCl, 0.003% LMNG, 0.0004% CHS, 0.001% GDN, 100 µM TCEP, 5 mM EDTA, 200 uM flag peptides. Eluted protein complex was concentrated and loaded onto Superdex 200 increase 10/300 GL column and peak fractions was collected and concentrated for further electron microscopy analysis.

Expression and purification of Gs heterotrimer, Nb35 and scFV16

Gs heterotrimer was expressed in Trichoplusia ni Hi5 insect cells (Invitrogen). Human Gαs was cloned in pFastbac1 vector, and N-terminal 6×His-tagged rat Gβ1, and bovine Gγ2 were cloned into pFastBac-Dual vector, and the viruses were prepared the same as GPR3. The cells were infected with both Gαs and Gβγ virus at a ratio of 10 :1 at 27 °C for 48 hours. The Gs heterotrimer was purified as previously reported. Briefly, cells were lysed in hypotonic lysis buffer and membrane was collected by centrifuge. Then it was resuspended in solubilization buffer containing 1% sodium cholate for 1 hour at 4 °C and centrifuge again to remove the cell debris. The supernatant was loaded onto Ni-NTA resin and after flow though the resin was extensively washed. During the wash steps the

detergent was changed from 1% sodium cholate to 0.08 % DDM. Then the Gs protein was eluted with buffer contain 250 mM imidazole and treated with lambda phosphatase at 4 °C overnight. Note that all the buffers need to supplemented with GDP and MgCl2 to maintain G protein activity. The next day Gs protein was concentrated, fast frozen in LN2 and stored at -80 °C.

Nanobody-35 (Nb35) was expressed in the BL21(DE3) E. coli strain. The construct is a kindly gift from Kobilka Lab (Stanford University). It was transformed to BL21 cells and the protein expression was induced by 1 mM IPTG at 18 °C overnight. Collected bacteria cells were sonicated and the protein was extracted and purified by general nickel affinity chromatography protocol as a small soluble protein. Elute protein was concentrated and loaded onto Superdex 200 increase 10/300 size exclusion column (GE). The peak fractions were collected and concentrated, fast frozen in LN2 and stored at -80 °C.

The scFv16 was purified as a secreted protein. The scFv16 sequence was cloned into pFastbac1 vector with a N- terminal GP67 secretion signal peptide and a C-terminal His tag. The baculovirus was prepared in the same way as GPR3. Hi5 insect cells were grown to a density of 2.5 million per ml and infected with virus at a ratio of 1:40. After 60 hours, supernatant was collected and loaded onto Ni-NTA resin. After flow though, the resin was washed by 20 mM Hepes pH7.5, 500 mM NaCl. Protein was eluted by 20 mM Hepes pH 7.5, 500 mM NaCl and 250 mM imidazole and was concentrated and then loaded onto Superdex 200 increase 10/300 size exclusion column (GE). The peak fractions were collected and concentrated, fast frozen in LN2 and stored at -80 °C.

GPR3-Gs-Nb35-scFV16 complex formation and purification

1 L Sf9 cell pellets infected with virus containing GPR3 were lysed in 60 ml lysis buffer 10 mM HEPES, pH 7.5, 1 mM EDTA, 4 mg/ml iodoacetamide, 2.5 µg/ml leupeptin, 0.16 mg/ml Benzamidine. Then the cells were centrifuged at 12,000g for 1 hour to collect the membrane. The membrane was resuspended in a buffer 20 mM HEPES, pH 7.5, 100 mM NaCl, 2 mM CaCl2, 4 mg/ml iodoacetamide, 2.5 µg/ml leupeptin, 0.16 mg/ml Benzamidine and then 10 mg Gs protein, 10 mM MgCl2 and 1 ul apyrase were added and incubated at 4 °C overnight. The protein complex was formed on the membrane and then 1% LMNG (NG310 Anatrace), 0.1% CHS (CH210, Anatrace) was added to solubilize the membrane. Extracted protein complex was further purified by M1 flag resin affinity chromatography. During wash steps, the buffer was exchanged to 20 mM HEPES, pH 7.5, 100 mM NaCl, 2 mM CaCl2, 0.003%

LMNG, 0.0004% CHS, 0.001% GDN (GDN101, Anatrace). The protein complex was finally eluted by elution buffer 20 mM HEPES, pH 7.5, 100 mM NaCl, 0.003% LMNG, 0.0004% CHS, 0.001% GDN, 100 µM TCEP, 5 mM EDTA, 200 uM flag peptides. Eluted protein complex was concentrated and loaded onto Superose 6 increase 10/300 GL column and peak fractions was collected and concentrated for further electron microscopy analysis.

Cryo-EM sample preparation and data collection

The amorphous alloy film49 (CryoMatrix nickel titanium alloy film, R1.2/1.3, Zhenjiang Lehua Electronic Technology Co., Ltd.) was glow discharged at Tergeo-EM plasma cleaner. 3 µL purified GPR3-Gs-Nb35-scFV16 complex sample was applied onto the grid and then blotted for 3 s with blotting force of 0 and quickly plunged into liquid ethane cooled by liquid nitrogen using Vitrobot Mark IV (Thermo Fisher Scientific, USA). Cryo-EM data were collected at the Kobilka Cryo-EM Center of the Chinese University of Hong Kong (Shenzhen), on a 300 kV Titan Krios Gi3 microscope. The raw movies were recorded by a Gatan K3 BioQuantum Camera at the magnification of 105,000, The pixel size is 0.83 Å. Inelastically scattered electrons were excluded by a GIF Quantum energy filter (Gatan, USA) using a slit width of 20 eV. The movie stacks were acquired with the defocus range of −1.2 to −2.0 micron with a total exposure time 2.5 s fragmented into 50 frames (0.05 s/frame) and with the dose rate of 20.6 e/pixel/s. The semi-automatic data acquisition was performed using SerialEM.

Cryo-EM data processing and model building

A total 4669 image stacks were collected and subjected for motion correction using MotionCor2[1]. Contrast transfer function parameters were estimated by CTFFIND4[2], implemented in RELION[3]. 2,971,163 particles were auto-picked and then subjected to 2D classification and Ab-initio reconstruction using cryoSPARC[4]. After 3 rounds of 3D classification with global angular search, 474,411 selected particles were further subjected to 3D classification with local angular search using RELION. Class 1 with Nb35 containing 119,361 particles was subjected to NU-refinement in cryoSPARC to yield a 3.1 Å map. Class 2-4 without Nb35 containing 355,050 particles were further classified using hetero-refinement in cryoSPARC. Eventually, 217,646 particles were selected and subjected to NU-refinement in cryoSPARC to yield a 3.0 Å map. Local resolution map was calculated using cryoSPARC.

The initial model of active-state GPR3 was built by SWISS-MODEL using the cannabinoid receptor 2 (PDB ID 6PT0) as a template. The coordinates of Gs, Nb35 and scFv16 were selected from the V2R-Gs (PDB ID 7KH0) and CB2-Gi-scFv16 (PDB ID 6PT0) structures. All models were docked into the EM density map using Chimera followed by iterative manual building in Coot[5] and refinement in Phenix[6]. The final model statistics was validated by Molprobity[7].

GTPase-Glo^{TM} assay

The GPR3-Gs complex use for GTPase-GloTM assay were purified as described above and frozen at -80 °C before use. The GTPase reaction was initiated by adding 0.5 uM GPR3-Gs protein complex in 5 μL reaction buffer (20 mM HEPEs, 100 mM NaCl, 0.02% LMNG, 1 mM MgCl2, 5 μM GTP, 5 μM GDP, with or without ligand) in a 384-well plate. The GTPase reaction was incubated at room temperature (22-25°C) for 2 hours. After incubation, 5 μL reconstituted 1xGTPase-GloTM Reagent (Promega) was added to the completed GTPase reaction, mixed briefly and incubated with shaking for 30 minutes at room temperature (22-25°C) to convert the remaining GTP into ATP. Then 10 μL Detection Reagent (Promega) was added to the system and incubated in the 384-well plate for 5-10 minutes at room temperature (22-25°C) to convert the ATP into luminescent signals. Luminescence intensity was quantified using a Multimode Plate Reader (PerkinElmer EnVision 2105) luminescence counter. Data were analyzed using GraphPad Prism 9.0.

Cell surface expression determination by flow cytometry

The transfected HEK293 cells were collected and washed with PBS. Next, the cells were blocked with 5% BSA at room temperature for 15 minutes, followed by incubation with anti-Flag antibody (1:100) in PBS containing 1% BSA at 4°C for 1 hour. Afterward, the cells underwent two additional wash steps and were then incubated with anti-mouse Alexa-488-conjugated secondary antibody (1:300, Beyotime) in PBS containing 1% BSA at 4°C in the dark. Subsequently, the cells were washed two times with PBS, and finally, they were resuspended in 200 µl PBS for detection in the BD Accuri™ C6 Plus flow cytometer. Approximately 10,000 cellular events were counted for each sample, and the fluorescence intensity data were collected. The data were analyzed using GraphPad Prism 9.0. and normalized to wild type (WT) GPR3.

cAMP-Glo Sensor assay

GloSensor cAMP assay was performed as a technical manual (Promega). Briefly, the wild type (WT) GPR3 and the indicated mutants were cloned into the pcDNA 3.1 vector with an N-terminal HA signal peptide, Flag epitope and a 3C protease site, and co-transfected with 22F cAMP Plasmid into HEK293T in 6-well cell dishes. After 24 hours, cells were seeded into 96-well plates in CO_2 independent medium and equilibrated with the GloSensor cAMP reagent. The cells were incubated for 1 hour at 37 °C and 1 hour at room temperature. Serially diluted compounds were added to plates and the luminescence signals were countered by a microplate reader (PerkinElmer). Data were analyzed using GraphPad Prism 9.0.

Mass Spectrometry

LC-MS/MS analysis was conducted using Agilent 6549 triple quadrupole mass spectrometer connected with Agilent 1290 LC system. Chromatographic separation was achieved on an ACQUITY UPLC BEH C18 column (100mm×2.1 mm, 1.7 µm) at 50 °C. The mobile phase consisted of 10 mM ammonium acetate, 0.2 mM ammonium fluoride in 9:1 water/methanol (A) and 10 mM ammonium acetate, 0.2 mM ammonium fluoride in 2:3:5 acetonitrile/methanol/isopropanol (B) at a flow rate of 0.3 ml/min. The gradient of mobile phase B was 70% in 1min，70% to 86% in 2.5 min, held at 86% for 6.5min, then 86% to 100% in 1min, held at 100% for 6 min, then 100% to 70% in 0.1min, held at 70% for 1.9 min. The sample volume injected was 3 µL. Each infection has 3 replicates.
Mass spectrometer operating in positive ion mode using the following settings: Sheath gas temperature 200 °C, Sheath gas flow 11 L/min, Capliary 3.0 kV, Gas temperature 200 °C, gas flow 14L/min, Nebulizer 20 psi. Sheath Gas Temp Compounds were measured by multiple reaction monitoring (MRM) with optimized instrumental parameters. Quantifier MRM transitions (m/z) of Target Compounds: OEA 326.3/62.1: OA 282.3/41.1.

MD simulations

General system preparation

The following described simulations are based on the here reported active state GPR3 cryoEM. Missing residues in ICL3 were modeled with the natural amino acid sequence of GPR3 using the MODELLER software[8]. To avoid unnatural charges, the N- and C- Termini were capped with acetyl and N-methylamide groups. Titratable residues were left in their dominant protonation state at pH 7.0 except for $Asp^{2.50}$ being protonated

in the active structure. Additionally, a sodium ion proposed to stabilize the inactive state interacting with the allosteric site around Asp$^{2.50}$ was modeled into the apo state model by aligning the GPR3 receptor model to a high-resolution inactive structure of the adenosine A_{2a} (PDB-ID 5IU4[9]) containing the said sodium ion[10]. Parameter topology and coordinate files were generated using the tleap module of the AMBER18 program package[11]. Subsequent energy minimization was performed using the PMEMD module of AMBER18 by applying 500 steps of the steepest decent algorithm followed by 4500 steps of the conjugate gradient algorithm. Orienting of the protein inside a pre-equilibrated membrane of dioleoyl-phosphatidylcholine (DOPC) lipids was done by aligning the prepared receptor to the orientation of proteins in membranes (OPM[12]) model structure of β_2AR (PDB-ID 3SN6[13]). Embedding of the protein into the membrane was done using the g_membed GROMACS module[14]. Inserting sodium and chloride ions to the waterbox resulting in a 0.15 M NaCl solution ensured a physiological environment for the receptor. The now prepared simulation systems were energy minimized and equilibrated using the NVT ensemble at 310 K for 1.0 ns followed by the NPT ensemble for 1.0 ns with harmonic restraints of 10.0 kcal·mol^{-1} on the protein. In the NVT ensemble, the V-rescale thermostat was used. In the NPT ensemble the Berendsen barostat with a compressibility of 4.5×10^{-5} bar^{-1} was applied. The systems were further equilibrated for 25 ns with restraints on protein backbone atoms. Restraints were reduced step by step every 5.0 ns, starting with 10.0, going down to 5.0,1.0,0.5 and lastly 0.1 kcal·mol^{-1}.

Unbiased MD simulations of receptor ligand complexes

To reduce the overall system size and therefore enabling faster simulation the intracellular binding partner was omitted and the G protein interface of GPR3 restrained by applying harmonic restraints of 10 kcal mol^{-1} Å^2 to ensure a stable active conformation. In addition to the receptor preparation, ligand parameters were assigned via the AMBER module antechamber[11]. Ligand geometry optimization was done using Gaussian 16[15] at B3LYP/6-31G* level of theory and charges were calculated at HF/6-31Gs level of theory. Furthermore, atom point charges were assigned according to the RESP procedure[16]. Simulations were performed using GROMACS 2021.5[17]. The general AMBER force field (GAFF)[18] was used for the ligands.

Unbiased and metadynamic simulations of the apo state GPR3

Apo state simulations were prepared and conducted as described previously[19]. For this protocol the intracellular binding partner and co-crystallized ligand were omitted. Initially an unbiased MD production run was performed cumulating 10μs of simulation time using GROMACS2021.5[17]. The resulting trajectory was checked for 32 appropriate frames with different conformations of the key micro-switches. The selected frames were subject to a subsequent well-tempered multiple walker simulation[20,21] using GROMACS 2021.4 software patched with the PLUMED plugin[22]. Applying a bias potential to the distance between $R^{3.50}$ and $I^{6.34}$ (representing the TM3-TM6 distance) as a collective variable enabled a reconstruction of the free energy surface and allowed for extraction of a representative frame of the energetical minimum. Gaussian hills with a height of 0.239 kcal·mol^{-1} were applied every 1.0 ps. Hill width was set to 1.0 Å. Rescaling of the gaussian function was done with a bias factor of 25.

All productive simulations were performed with periodic boundary conditions using the lipid14 force field[23] for DOPC molecules, ff14SB[24] for protein residues, and the SPC/E model for water[25]. A time step of 2 fs with bonds involving hydrogen constrained using LINCS[26] was chosen. Long-range electrostatic interactions were calculated using the particle mesh Ewald (PME) method[27] with interpolation of order 4 and fast Fourier transform (FFT) grid spacing of 1.6 Å. Non-bonded interactions cut off was set to 12.0 Å.

Measurement of constitutive activity

For constitutive Gs signaling measurement, full-length human GPR3 and β2AR were inserted into the pCAGGS expression plasmid with the N-terminal haemagglutinin signal sequence followed by the FLAG epitope and the HiBiT tag flanked by with flexible linkers (MKTIIALSYIFCLVFA-DYKDDDDK-GGSGGGGSGGSSSGGG-VSGWRLFKKIS-GGSGGGGSGGSSSG). LgBiT (gene synthesized with codon optimization by GenScript) was inserted into the pET-28a (+) vector with an N-terminal flexible linker (GGGGSGGGGS) and expressed in E.coli and purified using Ni-NTA resin, as described elsewhere[28]. Constitutive Gs signaling was measured by an in-house modified GloSensor cAMP assay (Promega) and normalized by HiBiT-based surface expression analysis performed in parallel. HEK293 cells were harvested and suspended in Opti-MEM I Reduced Serum Medium (Thermo Fisher Scientific) at a cell concentration of 4 x 10^5 cells ml^{-1}, seeded in a 96-well white culture plate (80 μl per well) and placed in a CO_2 incubator. Transfection solution (per well in the 96-well plate hereafter) was prepared

by mixing 40 ng of a Glo-22F cAMP biosensor (gene synthesized with codon optimization by GenScript)-encoding pCAGGS plasmid and titrated volumes of the N-terminally HiBiT-tagged GPCR plasmid (from 0.2 ng to 8 ng; 2-fold or 2.5-fold titration) plus a balance of the empty pCAGGS plasmid (total plasmid volume of 28 ng), along with 0.2 μl of 1 mg ml^{-1} PEI and 20 μl Opti-MEM I Reduced Serum Medium. Transfection was performed on the same day as cell seeding and the cells were cultured for 1 day. For the GloSensor-based cAMP measurement, 20 μl of the conditioned media were removed and the cells were mixed with 20 μl of 12 mM D-luciferin potassium solution (FujiFilm Wako Pure Chemical) diluted in HBSS containing 0.01% BSA and 5 mM HEPES (pH 7.4) (assay buffer). For the HiBiT-based surface GPCR expression measurement, 20 μl of the conditioned media were removed and the cells were mixed with 20 μl of LgBiT (1:200 of the stock solution) and 50 μM furimazine diluted in the assay buffer. After 2h (GloSensor) or 30-min (HiBiT) incubation in the dark at room temperature, the luminescence of each well was measured by a microplate luminometer with an integration time of 0.4 s per well with 5 rounds of readings (Spectramax L, Molecular Devices). The luminescent counts were normalized to that of mock-transfected cells prepared in the same plate and expressed as a fold-change value. For each GPCR, surface expression (HiBiT signal) and cAMP level (GloSensor signal) were plotted and those in linear correlation were used to calculate a slope (expression-normalized cAMP level), which was shown in the graph.

Surface plasmon resonance (SPR) analysis

The interaction between wild-type monomer GPR3 protein and oleoylethanolamide (OEA) were measured by surface plasmon resonance experiment that using Biacore X100 system (Cytiva). Firstly, the optimal pH immobilization environment was determined using the acetates with various pH value according to protein pre-enrichment procedure. The purified wild-type GPR3 protein were immobilized to the CM5 sensor chip (Cytiva) at the optimal pH 4.5. Then the different concentrations of OEA were prepared in a running buffer containing 20 mM HEPES (pH 7.4), 100 mM NaCl, 0.01% LMNG and 5% (v/v) DMSO and injected as analytes. In this test, we set 60 s binding period, 60 s disassociation period and 20ul/min flow rate for detection. The ligand-receptor binding activity is measured in resonance units (RU) and the interaction is recorded and displayed as a sensorgram in real-time. The data was analyzed in the Biacore X100 system for calculation of binding affinity (KD).

References

1 Zheng, S. Q. *et al.* MotionCor2: anisotropic correction of beam-induced motion for improved cryo-electron microscopy. *Nat Methods* **14**, 331-332, doi:10.1038/nmeth.4193 (2017).

2 Rohou, A. & Grigorieff, N. CTFFIND4: Fast and accurate defocus estimation from electron micrographs. *Journal of structural biology* **192**, 216-221, doi:10.1016/j.jsb.2015.08.008 (2015).

3 Scheres, S. H. RELION: implementation of a Bayesian approach to cryo-EM structure determination. *Journal of structural biology* **180**, 519-530, doi:10.1016/j.jsb.2012.09.006 (2012).

4 Punjani, A., Rubinstein, J. L., Fleet, D. J. & Brubaker, M. A. cryoSPARC: algorithms for rapid unsupervised cryo-EM structure determination. *Nat Methods* **14**, 290-296, doi:10.1038/nmeth.4169 (2017).

5 Emsley, P., Lohkamp, B., Scott, W. G. & Cowtan, K. Features and development of Coot. *Acta crystallographica. Section D, Biological crystallography* **66**, 486-501, doi:10.1107/S0907444910007493 (2010).

6 Adams, P. D. *et al.* PHENIX: a comprehensive Python-based system for macromolecular structure solution. *Acta crystallographica. Section D, Biological crystallography* **66**, 213-221, doi:10.1107/S0907444909052925 (2010).

7 Chen, V. B. *et al.* MolProbity: all-atom structure validation for macromolecular crystallography. *Acta crystallographica. Section D, Biological crystallography* **66**, 12-21, doi:10.1107/S0907444909042073 (2010).

8 Sali, A. & Blundell, T. L. Comparative protein modelling by satisfaction of spatial restraints. *J Mol Biol* **234**, 779-815, doi:10.1006/jmbi.1993.1626 (1993).

9 Segala, E. *et al.* Controlling the Dissociation of Ligands from the Adenosine A2A Receptor through Modulation of Salt Bridge Strength. *J Med Chem* **59**, 6470-6479, doi:10.1021/acs.jmedchem.6b00653 (2016).

10 Miao, Y., Caliman, A. D. & McCammon, J. A. Allosteric effects of sodium ion binding on activation of the m3 muscarinic g-protein-coupled receptor. *Biophys J* **108**, 1796-1806, doi:10.1016/j.bpj.2015.03.003 (2015).

11 Case, D. A. e. a. AMBER 2018, University of California, San Francisco. (2018).

12 Lomize, M. A., Pogozheva, I. D., Joo, H., Mosberg, H. I. & Lomize, A. L. OPM database and PPM web server: resources for positioning of proteins in membranes. *Nucleic Acids Res* **40**, D370-376, doi:10.1093/nar/gkr703 (2012).

13 Rasmussen, S. G. *et al.* Crystal structure of the beta2 adrenergic receptor-Gs protein complex. *Nature* **477**, 549-555, doi:10.1038/nature10361 (2011).

14 Wolf, M. G., Hoefling, M., Aponte-Santamaria, C., Grubmuller, H. & Groenhof, G. g_membed: Efficient insertion of a membrane protein into an equilibrated lipid bilayer with minimal perturbation. *J Comput Chem* **31**, 2169-2174, doi:10.1002/jcc.21507 (2010).

15 Frisch, M. J. e. a. Gaussian 16 Rev. A.03. (Wallingford, CT, 2016).

16 Bayly, C. I., Cieplak, P., Cornell, W. D. & Kollman, P. A. A Well-Behaved Electrostatic Potential Based Method Using Charge Restraints for Deriving Atomic Charges - the Resp Model. *J Phys Chem-Us* **97**, 10269-10280, doi:DOI 10.1021/j100142a004 (1993).

17 M.J. Abraham, T. M., R. Schulz, S. Páll, J.C. Smith, B. Hess, and E. Lindahl. GROMACS: High performance molecular simulations through multi-level parallelism from laptops to supercomputers. *SoftwareX*, 19–25 (2015).
18 Wang, J., Wolf, R. M., Caldwell, J. W., Kollman, P. A. & Case, D. A. Development and testing of a general amber force field. *J Comput Chem* **25**, 1157-1174, doi:10.1002/jcc.20035 (2004).
19 Chen, G. *et al.* Activation and allosteric regulation of the orphan GPR88-Gi1 signaling complex. *Nature Communications* **13**, doi:10.1038/s41467-022-30081-5 (2022).
20 Raiteri, P., Laio, A., Gervasio, F. L., Micheletti, C. & Parrinello, M. Efficient reconstruction of complex free energy landscapes by multiple walkers metadynamics. *J Phys Chem B* **110**, 3533-3539, doi:10.1021/jp054359r (2006).
21 Barducci, A., Bussi, G. & Parrinello, M. Well-tempered metadynamics: a smoothly converging and tunable free-energy method. *Phys Rev Lett* **100**, 020603, doi:10.1103/PhysRevLett.100.020603 (2008).
22 consortium, P. Promoting transparency and reproducibility in enhanced molecular simulations. *Nat Methods* **16**, 670-673, doi:10.1038/s41592-019-0506-8 (2019).
23 Dickson, C. J. *et al.* Lipid14: The Amber Lipid Force Field. *J Chem Theory Comput* **10**, 865-879, doi:10.1021/ct4010307 (2014).
24 Maier, J. A. *et al.* ff14SB: Improving the Accuracy of Protein Side Chain and Backbone Parameters from ff99SB. *J Chem Theory Comput* **11**, 3696-3713, doi:10.1021/acs.jctc.5b00255 (2015).
25 Berendsen, H. J. C., Grigera, J. R. & Straatsma, T. P. The Missing Term in Effective Pair Potentials. *J Phys Chem-Us* **91**, 6269-6271, doi:DOI 10.1021/j100308a038 (1987).
26 Hess, B., Bekker, H., Berendsen, H. J. C. & Fraaije, J. G. E. M. LINCS: A linear constraint solver for molecular simulations. *Journal of Computational Chemistry* **18**, 1463-1472, doi:Doi 10.1002/(Sici)1096-987x(199709)18:12<1463::Aid-Jcc4>3.0.Co;2-H (1997).
27 Darden, T., York, D. & Pedersen, L. Particle Mesh Ewald - an N.Log(N) Method for Ewald Sums in Large Systems. *J Chem Phys* **98**, 10089-10092, doi:Doi 10.1063/1.464397 (1993).
28 Dixon, A. S. *et al.* NanoLuc Complementation Reporter Optimized for Accurate Measurement of Protein Interactions in Cells. *Acs Chemical Biology* **11**, 400-408, doi:10.1021/acschembio.5b00753 (2016).

Supplementary information, Fig. S1

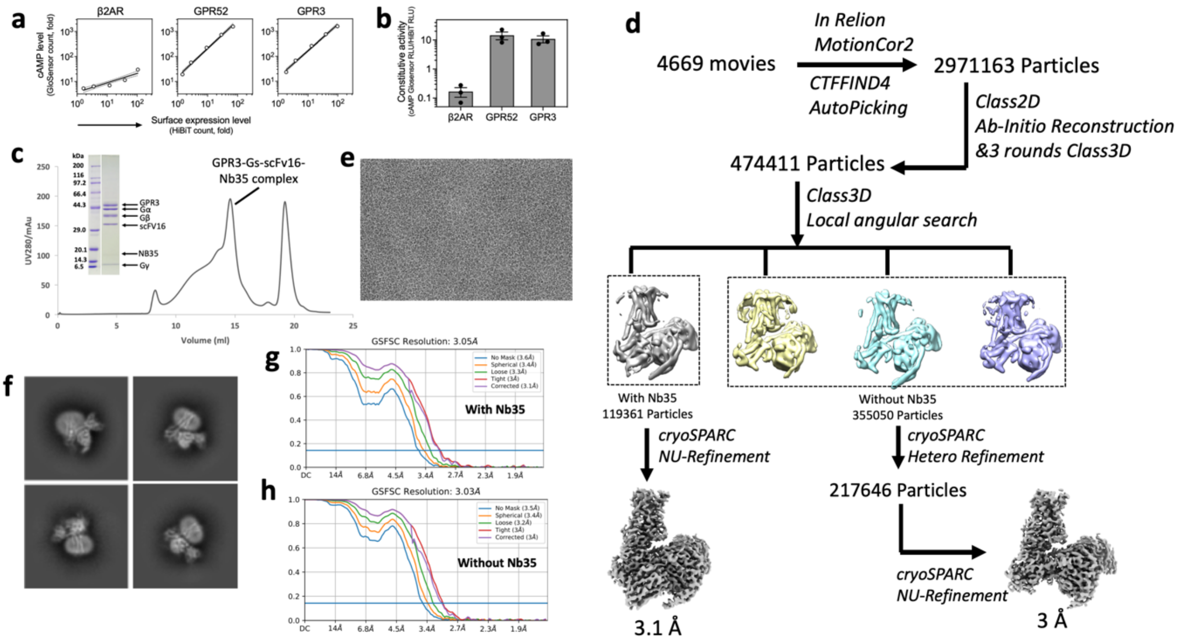

Supplementary information, Fig. S1. Sample preparation and cryo-EM data processing. a Representative data for surface expression level and cAMP level of N-terminally HiBiT-fused β2AR, GPR52 and GPR3 constructs. HiBiT signal and cAMP Glo-Sensor signal are shown as fold change over mock transfection. The GPCR plasmids were titrated by 4-fold and plotted in the graph with symbols and error bars representing mean and s.e.m., respectively, of 4 technical replicates from a single experiment. Lines and dotted lines indicate linear regression slopes and 95% confidence intervals, respectively. **b** Expression-normalized constitutive cAMP level of the indicated GPCRs. The expression-normalized cAMP levels were derived from the slope analysis in (**a**). Bars and error bars indicate mean and s.e.m., respectively, of 3 independent experiments (dots). **c** Size exclusion chromatography profile and SDS-PAGE of the GPR3-Gs-scFv16-Nb35 complex. **d** Cryo-EM data processing workflow. **e** Representative micrograph of the complex particles. **f** Representative 2D classification result. **g-h** Fourier shell correlation (FSC) curves with the estimated resolution according to the gold standard for the maps with (**g**) or without (**h**) Nb35.

Supplementary information, Fig. S2

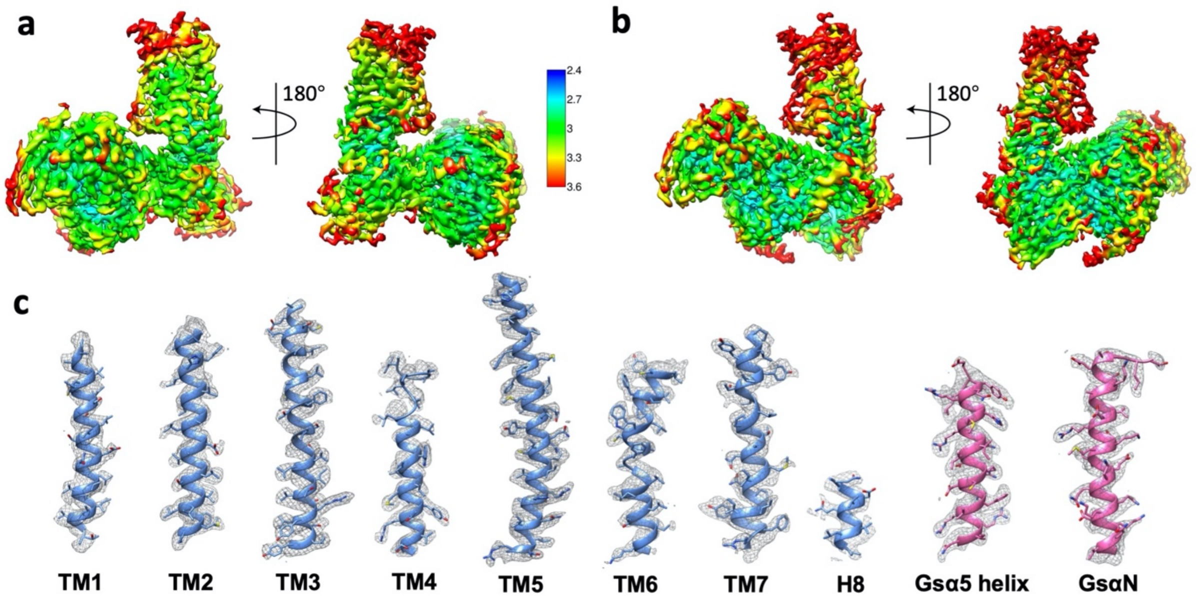

Supplementary information, Fig. S2. Cryo-EM density map and refined model. a Local resolution map without Nb35 viewed from two directions. **b** Local resolution map with Nb35 viewed from two directions. **c** Representative density maps and models for TM1-7 and H8 of GPR3 and the N-terminal and C-terminal α helices of Gαs (αN and α5).

Supplementary information, Fig. S3

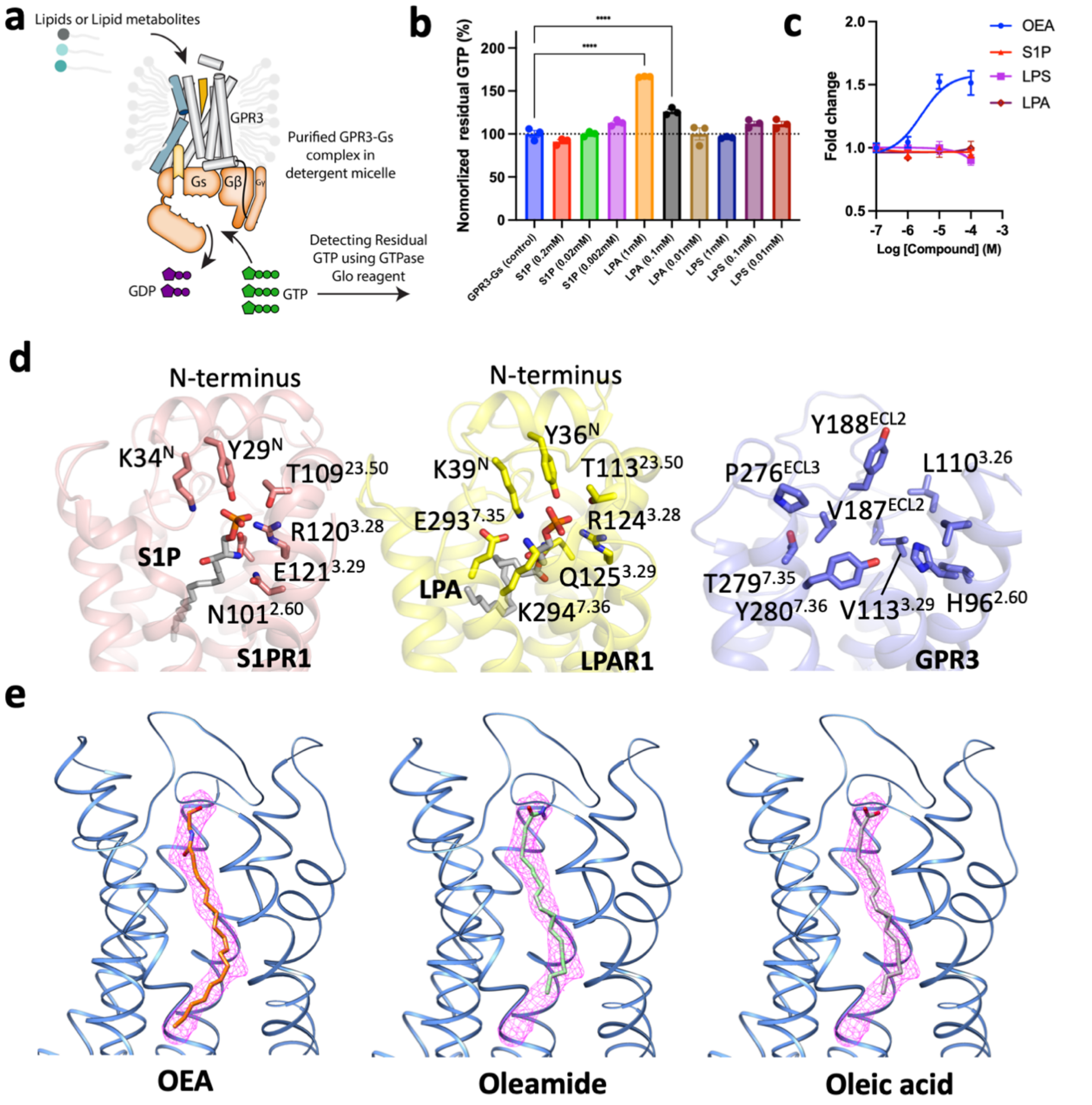

Supplementary information, Fig. S3. Functional evaluation of activation of GPR3 by different lysophospholipids and structural comparison. a Schematic diagram of in vitro GTP turn-over assay. Purified native GPR3-Gs signaling complex in detergent micelle was used to test different lipid-like molecules. **b** Activity of S1P, LPA and LPS on GPR3 measured by GTP turnover assay. The native GPR3-Gs without adding extra lipid-like molecules was set as control. Error bars denote mean±s.e.m. Statistical analyses were performed using the ordinary one-way ANOVA. ****$p<0.0001$. **c** Concentration response curves of OEA, S1P, LPA and LPS measured by cell-based cAMP Glo-Sensor assay. cAMP Glo-Sensor signal are shown as fold change over non-treated condition. Error bars denote mean±s.e.m. **d** Comparison of the

extracellular pocket of GPR3 with S1PR1 and LPAR1. The N-terminus of GPR3 is not resolved. **c** OEA has no effect on Gs as measured by GTPase Glo assay. Error bars denote mean±s.e.m. of three independent experiments. Statistical analyses were performed using the ordinary one-way ANOVA. **e** Modeling of different lipid molecules into the orthosteric density map: OEA, oleamide and oleic acid. OEA fits better into the density due to its longer shape.

Supplementary information, Fig. S4

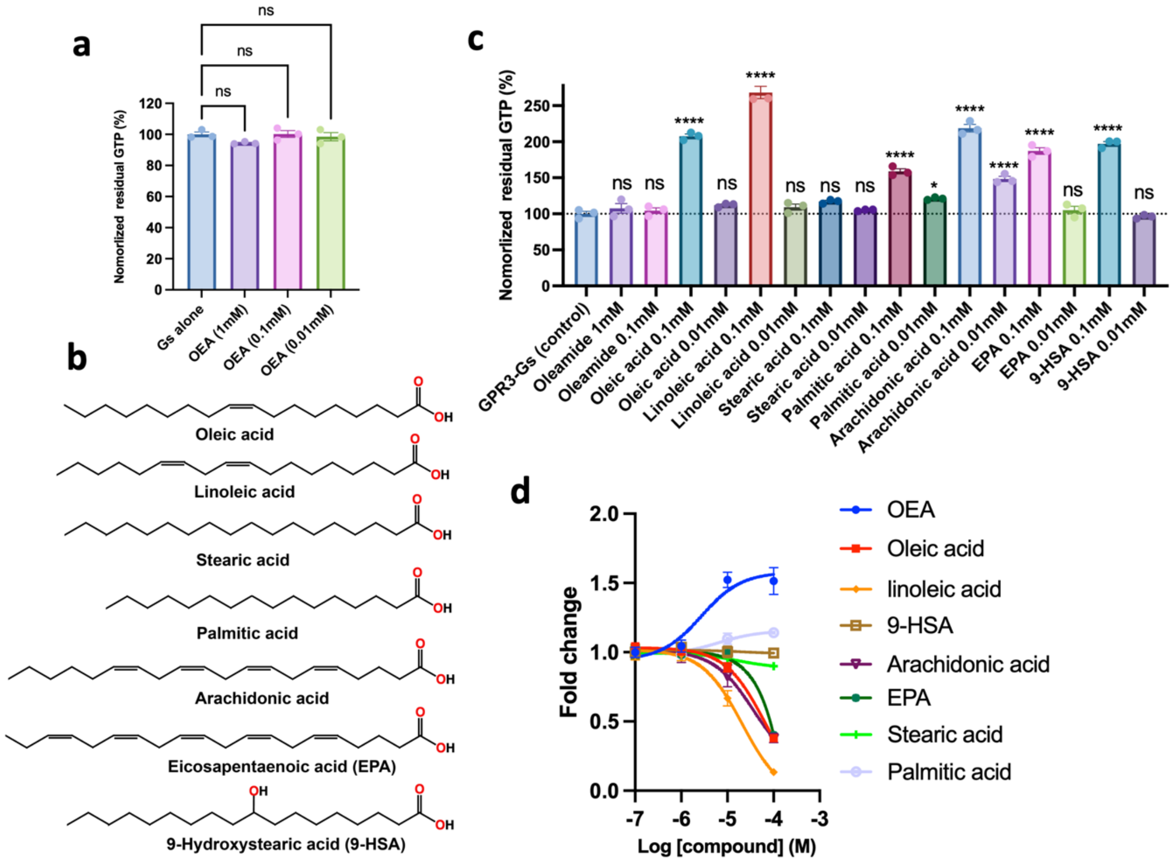

Supplementary information, Fig. S4. Functional evaluation of activation of GPR3 by free fatty acids. a Effect of OEA on Gs protein measured by GTP turn-over assay. **b** Chemical structures of free fatty acids. **c-d** Effects of free fatty acids on the activity of GPR3 measured by in vitro GTP turnover assay (**c**) and cell-based cAMP Glo-Sensor assay (**d**), respectively. Error bars denote mean±s.e.m. Statistical analyses were performed using the ordinary one-way ANOVA. ***p<0.001, ****p<0.0001, ns (not significant).

Supplementary information, Fig. S5

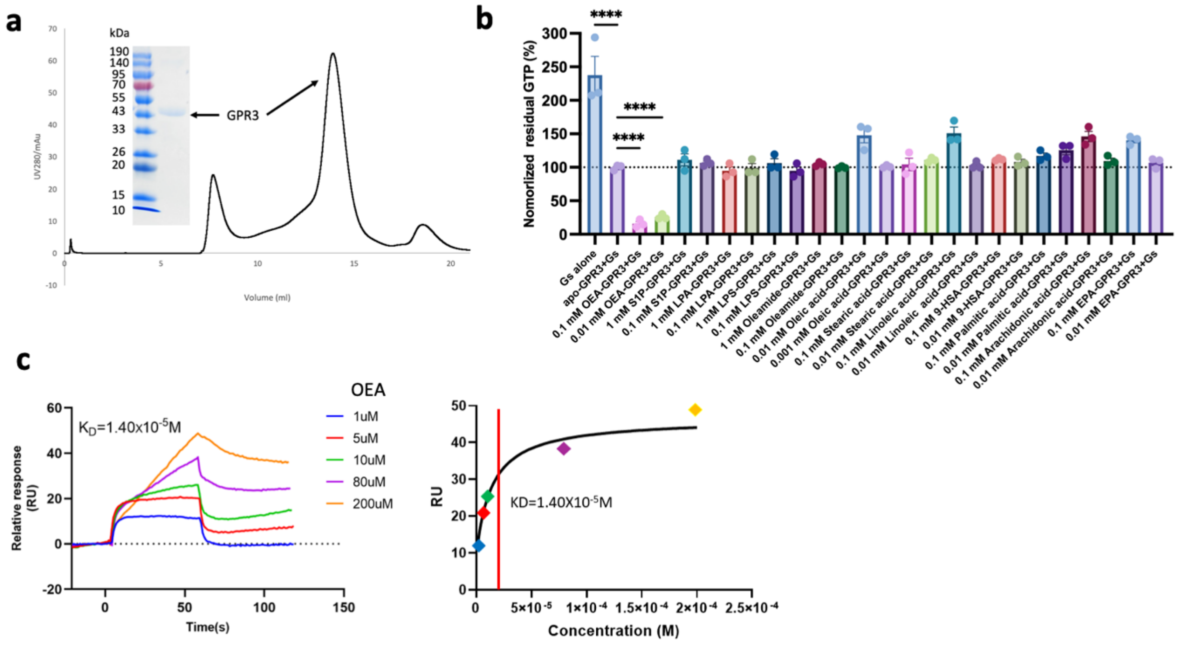

Supplementary information, Fig. S5. Functional evaluation of purified apo-form GPR3. a Size exclusion chromatography profile and SDS-PAGE of GPR3. **b** Activity of OEA, lysophospholipids and free fatty acids on GPR3 measured by in vitro GTP turnover assay using purified GPR3 and Gs proteiins in detergent micelles. Error bars denote mean±s.e.m. Statistical analyses were performed using the ordinary one-way ANOVA. ****p<0.0001. **c** Binding of OEA with GPR3 measured suing SPR. Sensorgram and saturation curve of titration of OEA on GPR3 immobilized on a CM5 chip. Sensogram was obtained by using a different concentration of OEA(left). The binding curves were fit to a steady-state affinity model to get KD value of 14 μM (right).

Supplementary information, Fig. S6

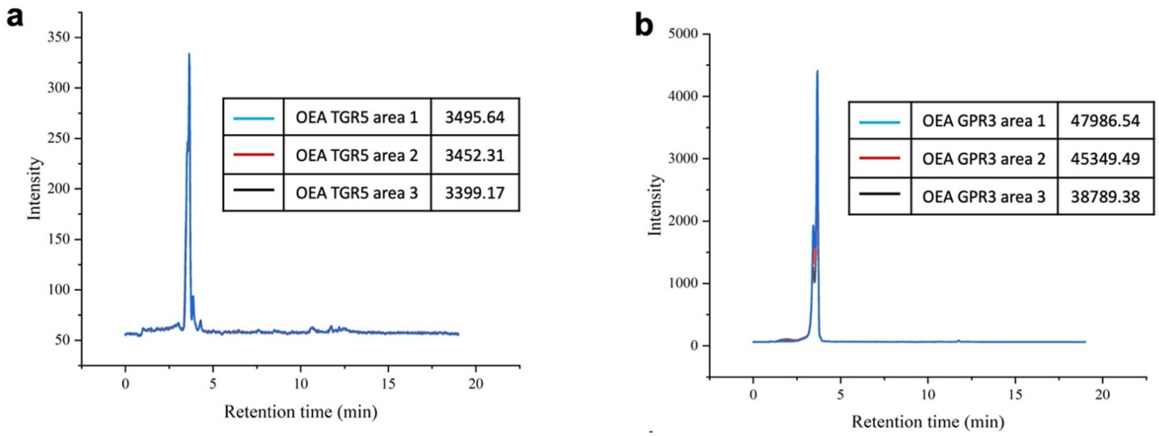

Supplementary information, Fig. S6. The LC-MS/MS analysis results. a The OEA peaks of TGR5-Gs sample. **b** The OEA peaks of native GPR3-Gs sample. The peaks for three independent expriments are overlaid together and the peak area for each experiment is shown in the inset table.

Supplementary information, Fig. S7

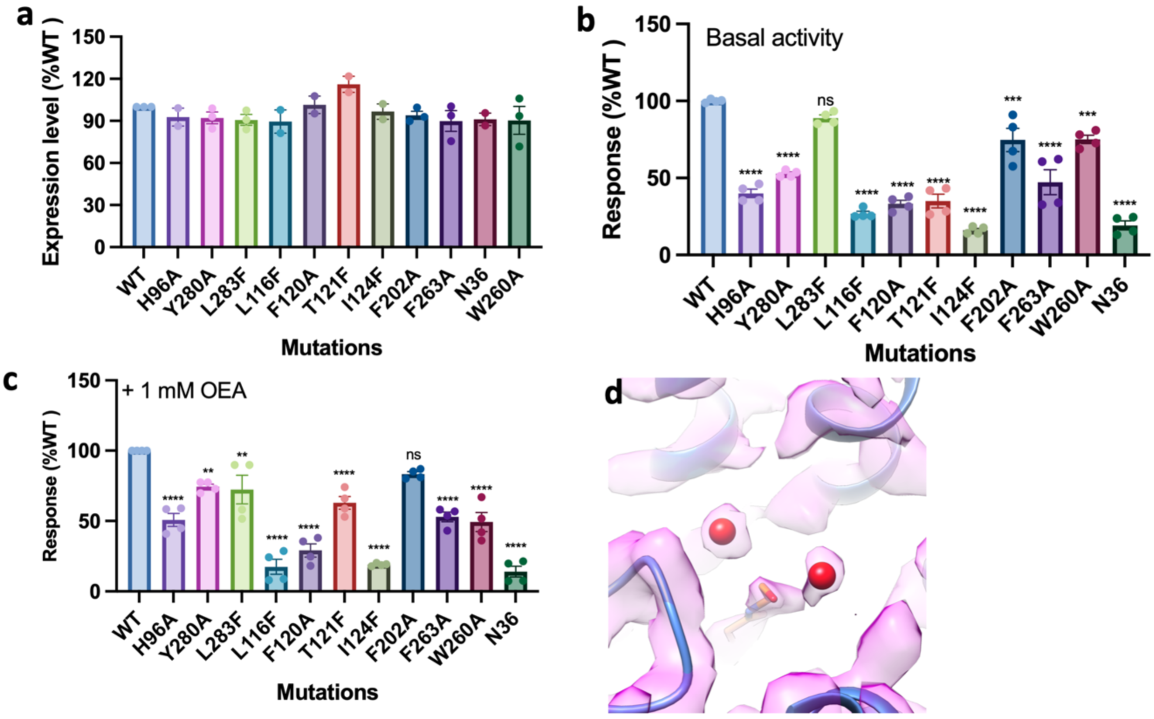

Supplementary information, Fig. S7. Mutagenesis analysis of ligand binding pocket. a Cell surface expression of mutant constructs measured by flow cytometry. **b-c** Effects of different mutations on the activity of GPR3 in the absence (**b**) or presence (**c**) of 1 mM OEA measured by cAMP Glo-Sensor assay. Error bars denote mean±s.e.m. Statistical analyses were performed using the ordinary one-way ANOVA. $^{**}p<0.01$, $^{***}p<0.001$, $^{****}p<0.0001$, ns (not significant). **d** The density map of the two water molecules on top of the ligand. The water molecules are shown in red spheres.

Supplementary information, Fig. S8

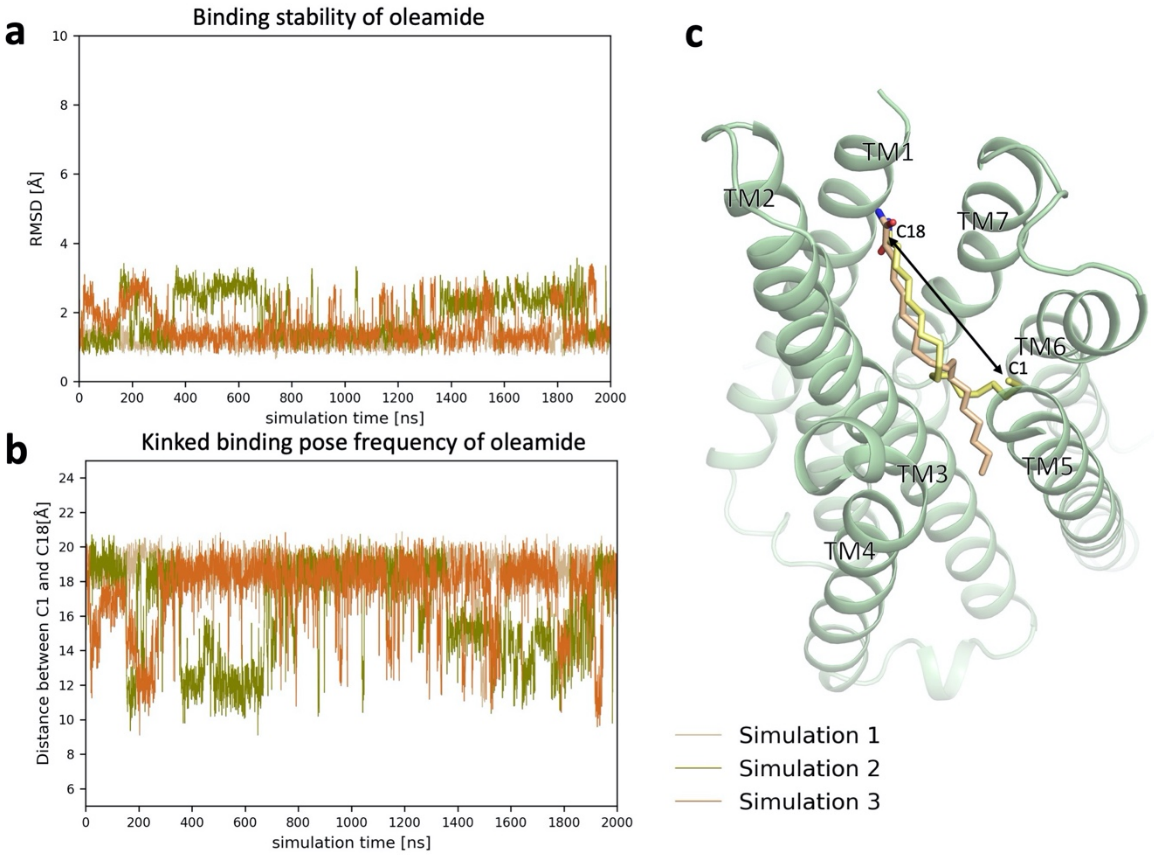

Supplementary information, Fig. S8. Molecular dynamics (MD) simulations of oleamide binding to GPR3. a The root-mean-square-deviation for oleamide in comparison to it's starting conformation. **b** The distance of C1 and C18 of the alkyl chain as a measurement for the frequency of a kinking motion. **c** Two representative MD-snapshots of GPR3 binding oleamide in either a straight or a kinked binding pose.

Supplementary information, Fig. S9

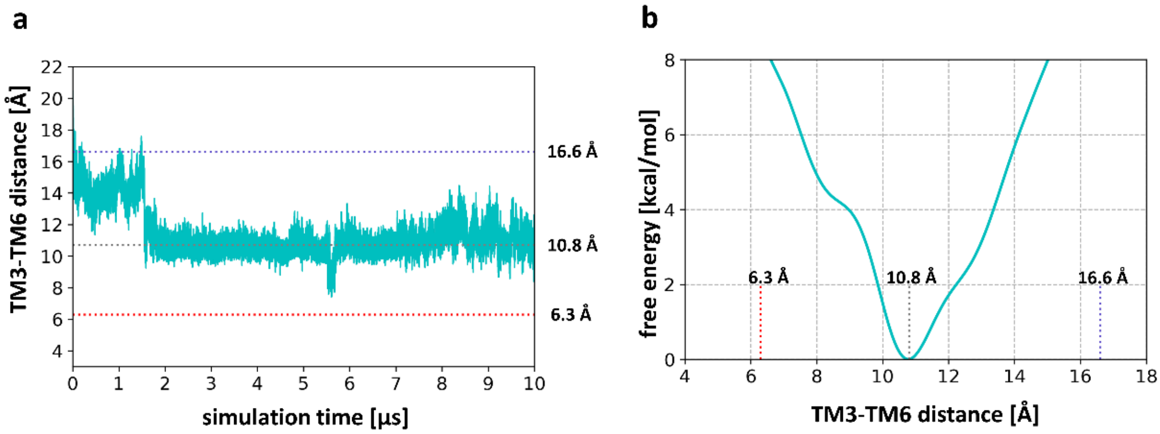

Supplementary information, Fig. S9. Unbiased and metadynamic MD simulations of apo state GPR3. a TM3-TM6 distance of GPR3 measured in an unbiased MD simulation over the course of 10μs after removing the intracellular binding partner **b** Free energy landscape along the TM3-TM6 distance of GPR3 after 6.72μs of metadynamics simulation showing an energetically minimum at a distance of 10.8 Å. Colored dashed lines indicating the TM3-TM6 distances of the different receptor activation states, with blue being the active cryoEM, gray being the apo state and red being the inverse agonist bound inactive state.

Supplementary information, Fig. S10

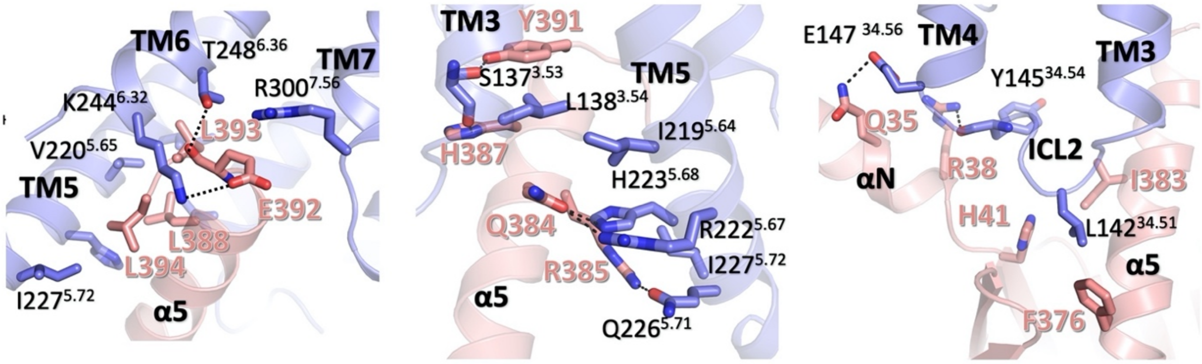

Supplementary information, Fig. S10. G-protein coupling interface. Cytoplasmic views of the GPR3 (slate) with the C-terminal α5 and αN helix of Gαs (red). H-bonds are shown as black dashed lines.

Supplementary information Table S1
Cryo-EM data collection, refinement and validation statistics.

**Data collection and processing**	**OEA-GPR3-Gs EMD-38015 PDB ID 8X2K**
Magnification	105,000
Voltage (kV)	300
Electron exposure (e^-/Å)	53
Defocus range (μm)	-1.0 to -2.0
Pixel size (Å)	0.83
Symmetry imposed	C1
Initial particle images (no.)	2971163
Final particle images (no.)	217646
Map resolution (Å)	3.03
FSC threshold	0.143
**Refinement**	
Initial model used (PDB code)	6PT0 and 7KH0
Map sharpening B-factor (Å)	-80
**Model composition**	
Non-hydrogen atoms	8570
Protein residues	1111
**B factor (Å)**	
Protein	49.67
Ligand	20
**R.m.s. deviations**	
Bond lengths (Å)	0.005
Bond angles (°)	1.073
**Validation**	
MolProbity score	1.74
Clashscore	7.12
**Ramachandran plot**	
Favored (%)	95.04
Allowed (%)	4.87
Disallowed (%)	0.09
